# Diurnally Entrained Anticipatory Behavior in Archaea

**DOI:** 10.1371/journal.pone.0005485

**Published:** 2009-05-08

**Authors:** Kenia Whitehead, Min Pan, Ken-ichi Masumura, Richard Bonneau, Nitin S. Baliga

**Affiliations:** 1 Institute for Systems Biology, Seattle, Washington, United States of America; 2 Division of Genetics and Mutagenesis, National Institute of Health Science, Tokyo, Japan; 3 Center for Genomics and Systems Biology, Courant Institute of Mathematical Science, Department of Computer Science, New York University, New York, New York, United States of America; 4 Department of Microbiology and Molecular and Cellular Biology, University of Washington, Seattle, Washington, United States of America; Universidad Miguel Hernandez, Spain

## Abstract

By sensing changes in one or few environmental factors biological systems can anticipate future changes in multiple factors over a wide range of time scales (daily to seasonal). This anticipatory behavior is important to the fitness of diverse species, and in context of the diurnal cycle it is overall typical of eukaryotes and some photoautotrophic bacteria but is yet to be observed in archaea. Here, we report the first observation of light-dark (LD)-entrained diurnal oscillatory transcription in up to 12% of all genes of a halophilic archaeon *Halobacterium salinarum NRC-1*. Significantly, the diurnally entrained transcription was observed under constant darkness after removal of the LD stimulus (free-running rhythms). The memory of diurnal entrainment was also associated with the synchronization of oxic and anoxic physiologies to the LD cycle. Our results suggest that under nutrient limited conditions halophilic archaea take advantage of the causal influence of sunlight (via temperature) on O_2_ diffusivity in a closed hypersaline environment to streamline their physiology and operate oxically during nighttime and anoxically during daytime.

## Introduction

The ability to anticipate impending environmental change(s) and mount a preparative response is crucial to the fitness of all organisms [1], [2]. Such preparatory behavior has been observed over a wide range of time scales (e.g. daily or seasonal variations) and is mediated via sensing, internalizing and subsequently recalling fluctuation patterns in the specific environmental factor(s) (EFs). Interestingly, such behavior can also result from the ability of biological systems (even microorganisms) to internalize and use reproducible interrelationships among EFs such that by sensing a change in one or few EFs (i.e. proxy variables) they are informed of impending changes in other EFs [3]. In other words, anticipatory or preparative behavior is a manifestation of gene regulatory networks that are appropriately structured to reproduce the cyclic nature and interrelatedness of EFs that have constrained their evolution [4].

In context of the diurnal cycle, anticipatory behavior appears widely throughout the eukaryotes and has been observed in some bacteria and is typical of organisms possessing circadian clocks [5]. However, photoresponsive anticipatory behavior is yet to be observed in archaea. The halophilic archaeaon *Halobacterium salinarum* was considered a prime candidate for LD entrainment of transcription owing to the presence in its genome of genes for four opsins, one putative cryptochrome and an ortholog of the bacterial clock component KaiC [6]. *H. salinarum NRC-1* uses light as a source of information for physical relocation towards favorable wavelengths of light or away from damaging radiation [7], [8], [9], [10], [11]. Under anoxic conditions it can use light-driven ion pumping by bacteriorhodopsin (bR) as means for producing ATP phototrophically [12], [13], [14]. Taken together with substantial evidence for light-mediated global gene regulation in this organism [15], [16], [17], these observations make a compelling case for investigating the feasibility of entraining global expression changes in *Halobacterium salinarum NRC-1* by prolonged culturing under diurnal 12h∶12h light∶dark (LD) cycles.

Here we present results of these experiments in which we detected free-running rhythms for at least 72 hours in up to 12% of all genes in *H. salinarum NRC-1* under constant darkness post-entrainment with 3 days of LD cycles. Remarkably, we observe that despite maintaining constant O_2_ during this experiment, a significant fraction of cycling genes are those that are also independently regulated by changes in O_2_ concentration [18]. This is interesting because O_2_ is another EF that has dominant influence on haloarchaeal physiology as a result of poor gas solubility in hypersaline environments. As such, we have previously demonstrated that a significant number of genes (at least 10%) in *H. salinarum NRC-1* are differentially regulated as a direct consequence of changes in O_2_ availability [18]. We conclude that *H. salinarum* can take advantage of coupled changes in sunlight and O_2_ such that it can use the L∶D cycle to anticipate higher levels of O_2_ during nighttime and lower levels during daytime. Given that entrainment of halobacterial physiology was best accomplished under nutrient limited condition we discuss this finding as a possible mechanism for maximizing resource utilization.

## Results and Discussion

**Figure 1:**
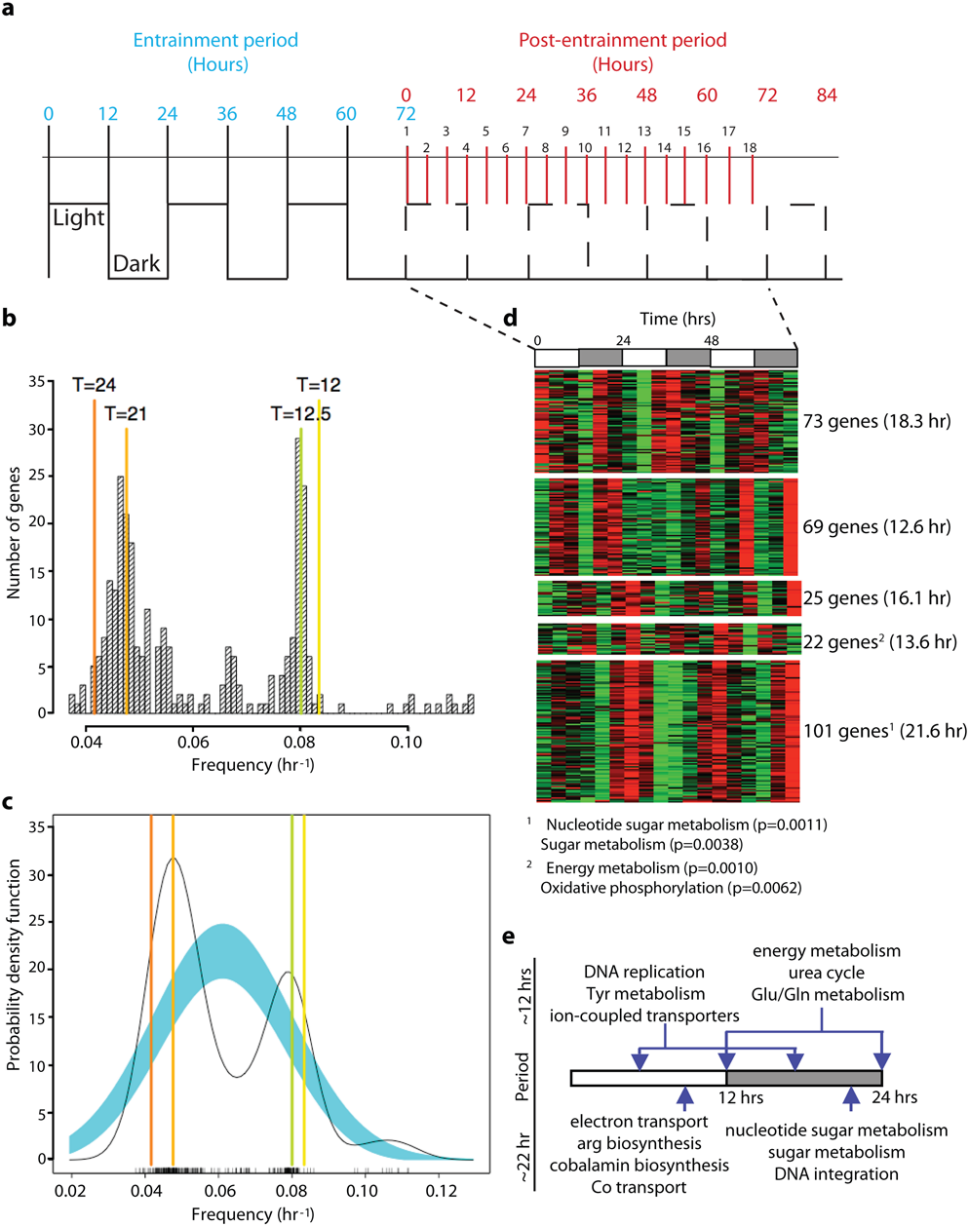
Discovery of diurnally entrained periodic gene expression in *H. salinarum NRC-1*. a, *H. salinarum NRC-1* cells were entrained with 3 days of 12∶12 LD and subsequently released into constant darkness. Total RNA was prepared from samples collected immediately post-entrainment (t = 0 hrs), every four hours until t = 60 hours. Two additional samples were collected at t = 64.5 hrs and t = 68.5 hrs. Culture conditions during sampling were frequently monitored and controlled (Supplementary Table S1). b, Frequency histogram of genes detected with periodic transcriptional changes (binned at intervals of 0.001 hr^−1^, p<0.2) using Lomb-Scargle analysis. c, Spectral density (black line) of the histogram in (a) shows two dominant frequencies of ∼12.5 and ∼21 hours; the blue swath shows data distribution of normally distributed gene expression changes. d, Five k-means clusters of periodic transcriptional changes of the 290 genes (from Experiment A) in (a, b) are visualized as a heatmap [average period is shown in parentheses and overrepresented GO or KEGG physiological functions (p<0.01) are also indicated]. The phasing of the diurnal L∶D cycle is indicated at the top of the heatmap with alternating white and shaded rectangles, respectively. e, Phase alignment of periodic gene expression changes shows co-induction of related cellular functions according to the diurnal cycle. DNA replication, tyrosine metabolism and ion-coupled transporters were upregulated during the middle of the light and dark phase with a period of 13.6 hours. Transcription of genes encoding components of NADH dehydrogenase (*ndhG3* and *ndhG4*), cytochrome oxidase (*coxB*), the urea cycle and glutamine-glutamate metabolism peaked at the transitions from one phase to the next. Finally, nucleotide sugar metabolism, general sugar metabolism, and DNA integration were maximally induced during the latter half of the dark phase.

We investigated possible diurnal entrainment of gene expression in *H. salinarum NRC-1* by subjecting cultures at various cell densities (Supplementary Table S1) to 72 hours of light∶dark (L∶D) changes on a 12∶12 hour cyclic schedule. Cells were harvested over 3 or 4 hour intervals for up to 75 hours in continuous darkness post-entrainment (Fig 1a and Supplementary Table S1). The cell pellets were flash frozen and subsequently processed for transcriptome analysis using whole genome microarray hybridization [16], [19]. The duration of each experiment, sampling frequency and cell densities over which the experiments were conducted (as estimated by optical density (OD) at 600 nm) are reported in Supplementary Table S1.

The resulting microarray data (Geo accession number: GSE15282) were analyzed for periodic expression patterns using the Lomb-Scargle (LS) method [20] (see methods for details). The LS analysis makes use of a least squares fit of sinusoidal curves to a given time series, and thus does not require evenly spaced data and is tolerant to missing data points [21]. The null distribution for the periodogram was also derived to determine statistical significance (p-value) of detecting oscillatory gene expression patterns [22]. The application of this analysis to 5 extended time courses (3 experimental and 2 controls with durations up to 75 hours with a 3–4 hour sampling frequency) allowed us to investigate oscillatory expression patterns with periods ranging from 6 hrs to >30 hrs. A gene was considered to have oscillatory behavior if a periodic pattern was detected in its expression with a p-value<0.2 in its respective LS periodogram (Figure 1b and Supplemental Figure S1). Consistent with the 3-day 12∶12 L∶D entrainment regimen, statistically significant periodic expression patterns with dominant periods of ∼13.0 hrs or ∼21 hrs were detected in a total of 290 genes (∼12.1% of the genome) in Experiment A and 230 genes (9.6% of the genome) in Experiment B (Fig 1c, Supplementary Figure S1). When expanded to include transcriptionally-linked genes within operons [23] (Koide et al., submitted to *Mol Sys Bio*) this represents potential periodic transcription of up to 636 genes in Expt A (27%) and 460 genes in Expt B (19%). An overlap of 167 genes between these gene-sets demonstrated significant reproducibility across the two experiments (p<10^−8^). Significantly, periodic gene expression with either of the two dominant frequencies was not observed at a lower cell density (OD_600_<0.4) (this is discussed further below), in control cultures that received no entrainment but were otherwise processed identically; or after shuffling/randomization of the expression-matrices (Supplementary Figure S1).

**Figure 2:**
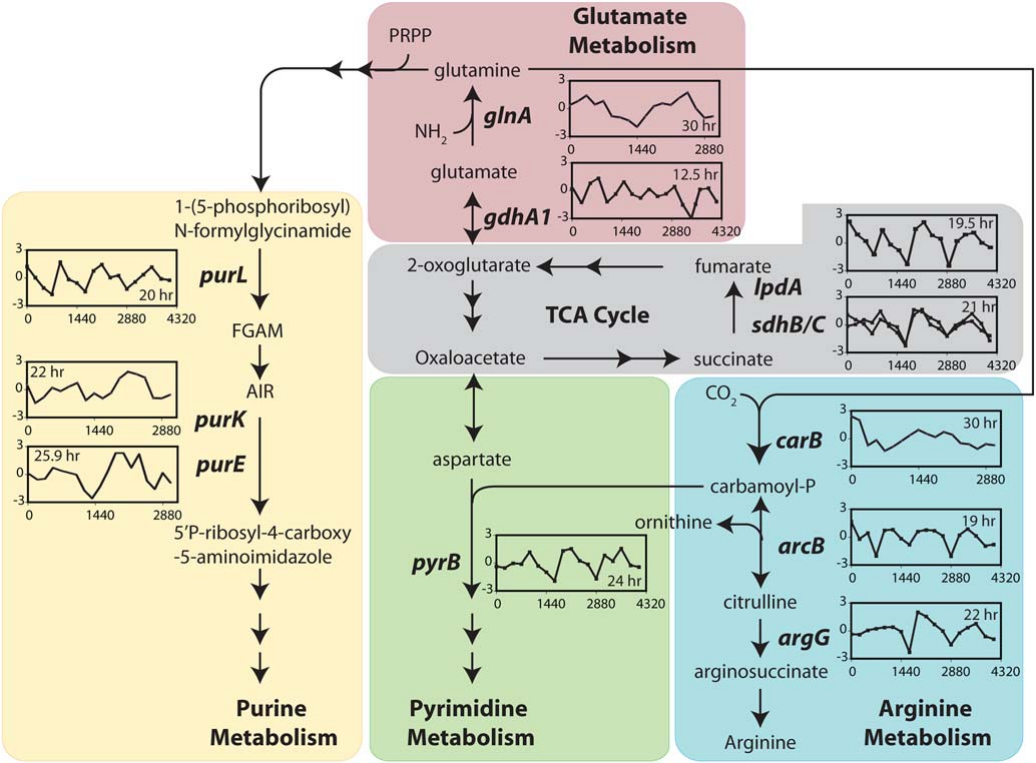
Periodic expression of genes in five linked processes. Integrated analysis of transcriptional changes from a physiological context identified periodic expression of genes encoding key steps in energy production (TCA cycle and arginine metabolism), C- and N- assimilation (glutamate and arginine metabolism) and nucleotide biosynthesis. The inset graphs show transcriptional profiles (log_10_ ratios) of gene with a specific period.

Genes with significant periodic expression patterns were further investigated in context of cellular physiology. This identified several classes of expression profiles, each with a distinct period and phasing and several with significant over-representation of diverse function categories (Fig 1d–e). This preliminary integrated analysis demonstrated the diurnal synchronization of a large number of linked enzymatic steps including key steps in the synthesis of nucleotides (Figure 2, Supplementary Figure S1B and Supplementary Table S2). Moreover, it was possible to phase-align several classes of oscillatory gene expression changes with the L∶D cycle (Fig 1e). This revealed that related cellular processes align well with respect to patterns of co-induction within the entrained transcriptional program.

**Figure 3:**
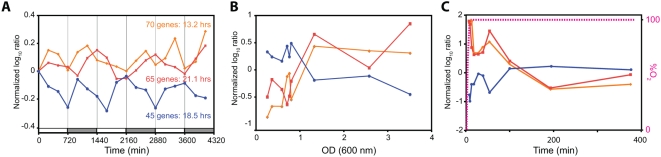
Entrained genes are directly linked to the oxygen and growth response in *H. salinarum NRC-1*. Three classes of average mRNA profiles for 180 of the 290 genes detected as cyclers in Experiment A. Expression profiles in all three panels are color-matched to indicate transcript profiles for the same three sets of genes over the LD cycle (A), in response to oxygen (B) and during growth in a batch culture (C). In panel A The period of oscillations in transcription upon entrainments is indicated as is the L∶D cycle (open∶grey boxes). Average transcript level changes in the same three groups of genes are plotted over the course of the growth curve for *H. salinarum NRC-1* (data from Facciotii et al. submitted). (C) The transcriptional response of these genes to sudden inflow of O_2_ after >6 hrs of anoxia [O2 levels are shown as a magenta dotted line (see secondary y-axis)] (Schmid et al. 2007).

Interestingly, diurnal entrainment of gene expression was maximally observed above a cell density (OD_600_>0.4) (Supplementary Figure S1) at which *H. salinarum NRC-1* is known to undergo a large physiological transition that involves the differential regulation of over 63% of all genes (Facciotti et al., submitted to *J. Bact*) through diverse mechanisms including activation of a large number of alternate promoters, terminators and putative ncRNAs (Koide et al., submitted). Not surprisingly, transcription of a significant fraction of cycling genes is also independently induced at this growth phase (Experiment A: p = 7×10^−5^; Experiment B: p = 3.7×10^−7^). This growth-phase dependent phenomenon results from exhausted nutritional resources including decreased oxygen carrying capacity in the medium - conditions akin to those in the natural environment of *H. salinarum NRC-1* [24] (Facciotti et al., submitted). Consistent with this observation, the 135 transcripts that are both periodically induced upon diurnal entrainment (including 70 genes with peak expression during daytime) and also independently upregulated at high cell density are significantly enriched for anoxic functions [18] (Figure 3, Supplementary Table S3) [18]. Surprisingly, the converse was also true - 45 transcripts that are typically downregulated at this growth phase and also independently repressed by a decrease in oxygen availability were also diurnally entrained with maximal expression during nighttime [18] (Fig 3; Supplementary Table S3). Remarkably, the distinct partitioning of periodic transcriptional changes in oxic and anoxic genes continues for at least 72 hours post-entrainment (Fig 3A). This clear split in oscillatory behavior of genes associated with oxic and anoxic functions strongly suggests synchronization and entrainment of oxygen-responsive physiologies according to the L∶D phase. Taken together these results demonstrate that nutrient and oxygen-limited conditions are the most conducive to entrainment with L∶D cycles – indicating perhaps the importance of synchronizing gene expression for efficient resource utilization under such conditions. Again, this periodic switching between oxic and anoxic physiologies was observed post-entrainment with the L∶D cycle, in constant darkness, and in culture conditions that were controlled to maintain constant dissolved oxygen (Supplementary Table S1). However, one could argue that natural oxygen consumption during aerobic growth and the subsequent adaptive shift to anaerobic physiology might have induced spontaneous cycling of oxic and anoxic gene expression similar to a phenomenon observed during continuous culturing of yeast [25]. We can rule out such a phenomenon because control experiments that were conducted simultaneously and at the same cell density did not result in oscillatory expression of oxic and anoxic physiology genes. Thus, we conclude that the 12∶12 L∶D entrainment indirectly induced cycling of oxygen-related physiologies and speculate that this might be an outcome of a natural relationship between changes in light and oxygen that has been internalized by *H. salinarum NRC-1*. This was initially intriguing because in most aquatic environments the direct physical coupling between light and oxygen via temperature is often confounded by diverse hydrological (river inflow, tides, rainfall, etc.) and biological (e.g. the balance between photosynthesis and respiration) phenomena [26], [27]. Further investigation into the physical characteristics of the natural hypersaline environment of halophilic archaea provided clues into the potential implication of light-mediated entrainment of oxygen-associated physiologies.

Extreme haloarchaea such as *H. salinarum NRC-1* thrive in closed ponds or terminal lake systems (such as the Great Salt Lake or the Dead Sea) with salinities in excess of 100–150 g salt L^−1^ [28]. Oxygen solubility is extremely poor at such high salinities and, not surprisingly, in addition to aerobic respiration most halophilic organisms also require alternate means of energy production such as phototrophy, denitrification and other dissimilatory processes [29], [30], [31], [32]. Adaptive responses that enable efficient conditional switching between these varied modes of energy production are critical for the energetically expensive lifestyle of halophilic organisms [16], [24], [33], [34], [35], [36]. For instance, in these environments, temperature and salinity are generally considered to be the dominant parameters influencing dissolved oxygen content [37] as biological primary production (photosynthesis) is greatly reduced [38], [39]. The physicochemical dependence of O_2_ concentration on temperature and salinity is well known [40], [41]. Notably, concentration of dissolved O_2_ in water drops as its temperature goes up; the solubility of O_2_ at 0°C is about twice its solubility at 30°C. Furthermore, there is evidence for an average diurnal cycle of 1–2°C in surface temperature of Great Salt Lake, a prototypically closed hypersaline ecosystem, with lower temperatures at nighttime [42]. Hence, higher oxygen levels are generally expected at nighttime relative to the warmer daytime period. Our data suggests that this physicochemical relationship between light and oxygen in the natural closed hypersaline environment has been imprinted onto the regulatory architecture of indigenous organisms such as *H. salinarum NRC-1*. In other words, under nutrient limited conditions halophilic archaea take advantage of this relationship to streamline their physiology by anticipating present and future linked changes in oxygen availability and operate oxically during nighttime and anoxically during daytime.

While such anticipatory behavior has been observed over shorter time scales [3], this study shows sustained oscillations in oxic/anoxic transitions over longer time scales through several cell divisions even after the L∶D stimulus is removed and the cells are maintained under constant conditions. Large families of haloarchaeal regulatory proteins (signal transducers and TFs) with physically linked domains for sensing light and oxygen are further evidence of tight coupling between these environmental factors and the biological architecture of the gene regulatory networks [6], [29], [43]. Finally, the discovery of diurnal entrainment of gene expression in an archaeon also raises important questions regarding the origin of light-responsive clock mechanisms. This is because archaeal information processing machinery is assembled from components that share ancestry with eukaryotic (general transcription factors and RNA polymerase) and bacterial (sequence-specific transcription regulators) counterparts [44]. Furthermore, components of both bacterial [45], [46] and eukaryotic [47] clocks are encoded in its genome [6], [32]. Indeed, further detailed experimentation is necessary to ascertain precise phasing, temperature compensation, adaptability to different periods of entrainment etc. to ascertain the mechanistic underpinnings of this diurnal entrainment and its physiological implications. Nonetheless, our results demonstrate that even extremophilic archaea can use the diurnal day/night cycle to their advantage by anticipating future physicochemically linked changes in other EFs.

## Materials and Methods

### Culturing, sampling and RNA extraction

Wild type *Halobacterium salinarium NRC-1* was cultured from a single colony in Complete Medium (CM) [48]; at 37°C with shaking at 125 rpm (Innova Waterbath, NewBrunswick Scientific, Edison, NJ). Cells were incubated under entrainment conditions (12∶12 L∶D cycle; daylight was simulated with full spectrum light at 150 µE/m^2^/s) or in continuous darkness (control) for three to four days prior to sampling. Post-entrainment Samples (2 ml) were collected periodically (every 3–4 hours) for up to 72 hours in continuous darkness, under constant cell density. The cell density was maintained by replacing a fixed volume in the culture (typically 30 mls) with equivalent of fresh CM every 3–4 hours [49]. Comparative analysis with a similarly processed control culture discounted any unaccounted perturbations that were introduced by this periodic dilution. Cell pellets were harvested by centrifugation at 1600 rcf for 2 min, decanted, flash-frozen in liquid N_2_ and stored at −80° until RNA extraction. Total RNA was prepared using the Absolutely RNA kit (Qiagen, Foster City, CA, USA). RNA quality was examined by spectrophotometry and BioAnalyzer (Agilent, Santa Clara, CA, USA) analyses and DNA contamination was ruled out by PCR with 16S rDNA primers.

### Microarray Analysis

*H. salinarum NRC-1* microarrays were fabricated at the Institute for Systems Biology Microarray Facility. Each microarray slide contains a unique 70mer oligonucleotide for each of the 2400 genes spotted in quadruplicate at two spatially distinct locations. Labeling, hybridization and washing have been previously described [16]. Statistical significance of differential gene expression was determined using the maximum likelihood method [19]. All microarray data reported in the manuscript is described in accordance with MIAME guidelines.

### Frequency Analysis

To examine the relative periodicity of genes in the constant light and constant dark experiments we used the Lomb normalized periodogram to estimate the spectral power as a function of angular frequency [20], [50], [51], [52]. This method can be used to evaluate whether a given gene is periodic or is the result of noise or some other non-periodic process (a p-value associated with the significance of the each peak in the periodogram can be easily calculated). There are other methods that would allow us to calculate periodograms and statistically evaluate whether a signal was truly periodic [22], [50], [53]; we chose the Lomb periodogram in part because it does not require evenly sampled data. Further, obeying the Nyquist limit, the highest frequency allowed was 0.167 hr^−1^ (period = 6 hrs). For our analysis the lowest frequency detected was 0.033 hr^−1^ (period = 30 hrs). This allowed for detection of a 24 hour signal and also for the experiment duration to contain two full periods over which to detect.

The Lomb periodogram, P_N_(ω), is calculated as follows:
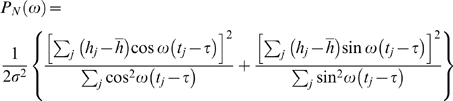
where mean and variance are calculated as per usual:
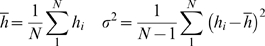
Tau is an offset that makes P_N_(ω) invariant to shifts in all time-points by a constant; tau is defined by the relation:
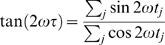
This offset removes phase from the calculation. The Lomb periodogram is analogous to least squares fitting of sins and cosines to our signal in the time domain.

The significance of periodicity of expression changes for each gene is then calculated as the probability that a peak in the periodogram with intensity greater than z is due to a random or non-periodic process [54]:
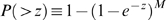
Where M is the effective number of independent frequencies sampled, which in our case is well approximated by N, the number of samples [55]. Thus, for each of the 2400 unique genes the analysis of a single time series resulted in a spectrogram and the significance of the maximum peak in that spectrogram.

### Data Integration and Visualization

Data were explored using the Gaggle and Firegoose framework for integrating diverse software tools and algorithms including Cytoscape, Data Matrix Viewer (DMV), KEGG, STRING, the R statistical package and MeV [56], [57].

## Supporting Information

Figure S1 A. Results from Lomb-Scargle analysis are presented as periodograms for each experiment described in Supplementary Table 1. Only genes with p<0.2 were considered to be cyclic in their expression pattern. Note that a strong banding pattern with p<0.2 is only observed in experiments A and B. B. Reproducibility of periodic transcriptional changes in 12 genes of diverse functions post-entrainment with three days of 12∶12 LD. Transcriptional changes over 48 hours of “memory” phase are shown along with putative functions.
(0.94 MB PDF)

Table S1 Experiment design, culturing parameters and sampling schedule.
(0.18 MB PDF)

Table S2 Genes with oscillatory gene expression profiles in Experiments A and B, period of oscilattion and significance.
(0.38 MB PDF)

Table S3 The number of genes and average period in each of the clusters presented in Figure 3 of the Experiment A.main text. The number of genes correlated to high or low oxygen (taken from Schmid et al. 2007) are also given.
(0.13 MB PDF)
