# Supplementary material for: Diurnally Entrained Anticipatory Behavior in Archaea
Source: PLoS One. 2009 May 8;4(5):e5485. doi: 10.1371/journal.pone.0005485 (PMC2675056; doi:10.1371/journal.pone.0005485)
Supplement: Figure S1 — A. Results from Lomb-Scargle analysis are presented as periodograms for each experiment described in Supplementary Table 1. Only genes with p<0.2 were considered to be cyclic in their expression pattern. Note that a strong banding pattern with p<0.2 is only observed in experiments A and B. B. Reproducibility of periodic transcriptional changes in 12 genes of diverse functions post-entrainment with three days of 12∶12 LD. Transcriptional changes over 48 hours of “memory” phase are shown along with putative functions. (0.94 MB PDF) [file pone.0005485.s001.pdf]

Figure S1A

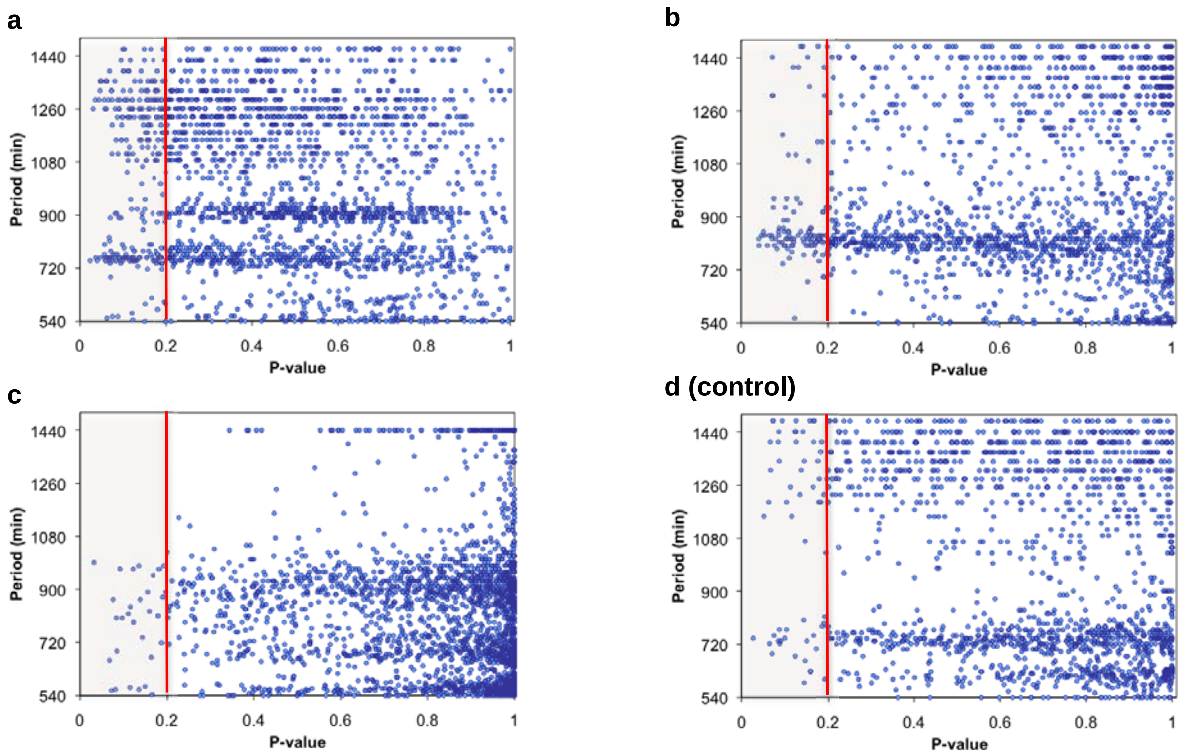

Figure S1A. Results from Lomb-Scargle analysis are presented as periodograms for each experiment described in Supplementary Table 1. Only genes with $p < 0.2$ were considered to be cyclic in their expression pattern. Note that a strong banding pattern with $p<0.2$ is only observed in experiments A and B.

Figure S1B

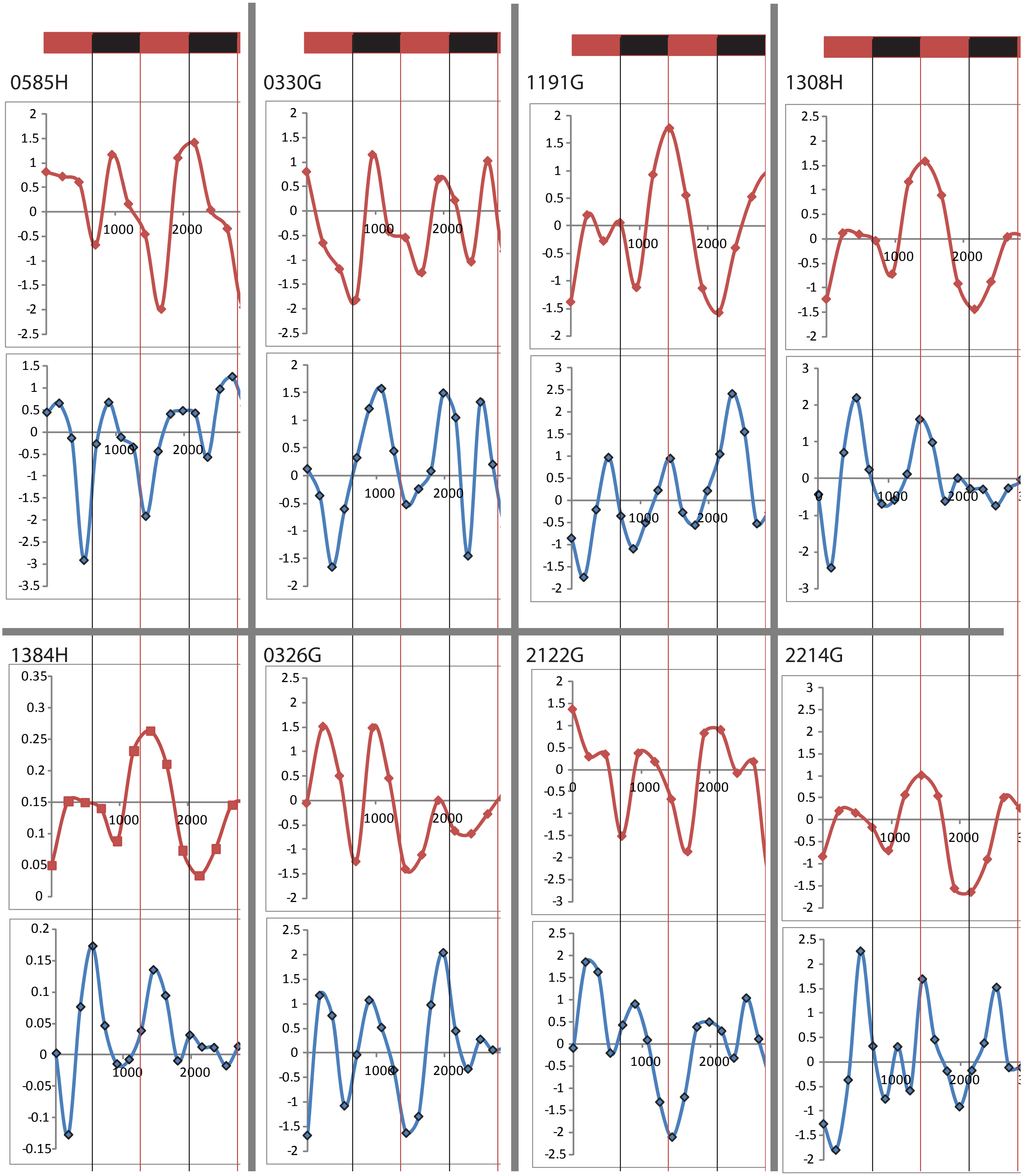

Figure S1B. Reproducibility of periodic transcriptional changes in 12 genes of diverse functions post-entrainment with three days of 12:12 LD. Transcriptional changes over 48 hours of "memory" phase are shown along with putative functions.

Figure S1B (continued)

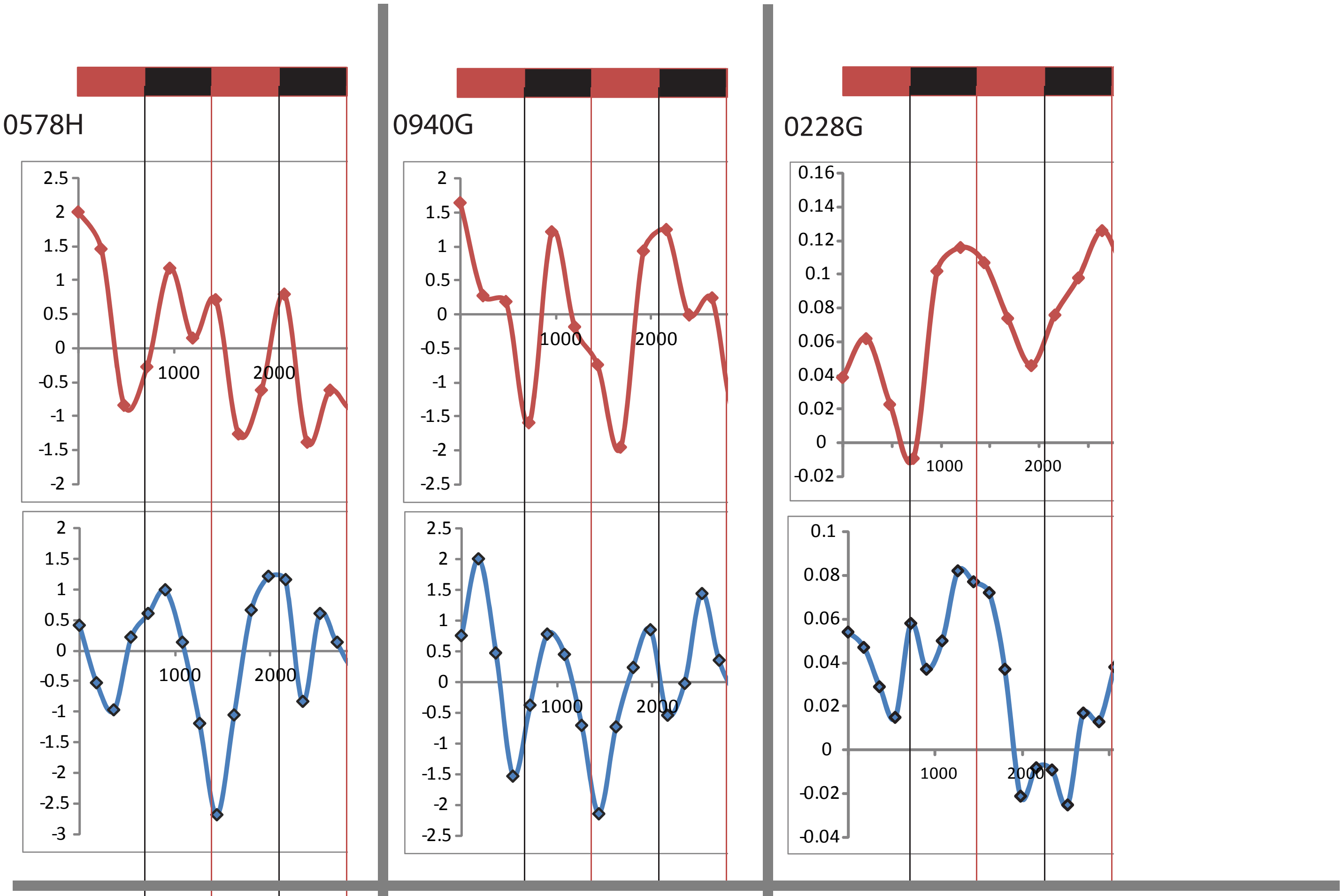

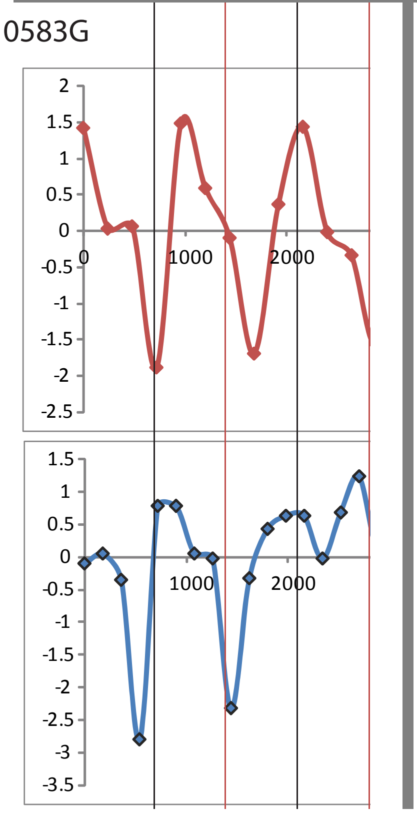

ORF Name	Gene Symbol	Function
VNG0228G	*guaAa*	*GMP synthase subunit A*
VNG0326G	*metS*	*Methionine-tRNA synthetase*
VNG0330G	*ppsA*	*Phosphoenolpyruvate synthase*
VNG0578H	*VNG0578H*	
VNG0583G	*cyb*	*Cytochrome b6*
VNG0585H	*VNG0585H*	
VNG0940Gm	*acs3*	*Acetyl-CoA synthetase (ADP forming)*
VNG1191Gm	*acd3*	*Acyl-CoA dehydrogenase*
VNG1384H	*VNG1384H*	
VNG2122G	*ilvE2*	*Branched-chain amino acid aminotransferase*
VNG2214G		*multidrug efflux pump*
VNG2430G	*thrC1*	*Threonine synthase*
VNG2431C	*VNG2431C*	
VNG2482G	*pstB1*	*Phosphate ABC transporter ATP-binding*

Figure S1B. Reproducibility of periodic transcriptional changes in 12 genes of diverse functions post-entrainment with three days of 12:12 LD. Transcriptional changes over 48 hours of "memory" phase are shown along with putative functions.
