# Supplementary material for: Diurnally Entrained Anticipatory Behavior in Archaea
Source: PLoS One. 2009 May 8;4(5):e5485. doi: 10.1371/journal.pone.0005485 (PMC2675056; doi:10.1371/journal.pone.0005485)
Supplement: Table S1 — Experiment design, culturing parameters and sampling schedule. (0.18 MB PDF) [file pone.0005485.s002.pdf]

Table S1. Experiment design, culturing parameters and sampling schedule.

**Experiment**	**Intensity (μE/m2/s)**	**shaking (rpm)**	**Temp (^oC)**	**OD min**	**OD max**	**dilution**	**duration (hrs)**	**#samples**
a	150	125	37	0.54	0.69	yes	68.5	18
b	150	125	37	0.38	0.68	yes	54	19
c	150	125	37	0.20	0.41	yes	75	25
control-1	150	125	37	0.38	0.68	yes	54	19
control-2	150	125	37	0.20	0.41	yes	75	25
