# Supplementary material for: Diurnally Entrained Anticipatory Behavior in Archaea
Source: PLoS One. 2009 May 8;4(5):e5485. doi: 10.1371/journal.pone.0005485 (PMC2675056; doi:10.1371/journal.pone.0005485)
Supplement: Table S2 — Genes with oscillatory gene expression profiles in Experiments A and B, period of oscilattion and significance. (0.38 MB PDF) [file pone.0005485.s003.pdf]

Table S2. Genes with oscillatory gene expression profiles in Experiments A and B, period of oscillattion and significance.

**Experiment A**					**Experiment B**				
**ORF**	**Gene**	**Function**	**Period**	**p-value**	**ORF**	**Gene**	**Function**	**Period**	**p-value**
VNG0006G	glmS	Glucosamine--fructose-6-phosphate aminotransferase [isomerizing]	1205.66	0.155	VNG0011C	VNG0011C	[+]	695.876	0.189
VNG0009G	graD2	Glucose-1-phosphate thymidylyltransferase	1290.909	0.0648	VNG0032H	VNG0032H	[+]	824.847	0.182
VNG0013C	VNG0013C	[+]	1354.77	0.197	VNG0041C	VNG0041C	[+]	1557.692	0.188
VNG0028C	VNG0028C	[+]	766.8	0.0809	VNG0047G	graD6	Glucose-1-phosphate thymidylyltransferase	1694.561	0.0673
VNG0029H	VNG0029H	[+]	1232.797	0.0963	VNG0058H	VNG0058H	[+]	1800	0.076
VNG0043H	VNG0043H	[+]	1154.819	0.192	VNG0066H	VNG0066H	potential transcriptional regulator, strong Rosetta match to E. coli transcriptional regulator	1646.341	0.0407
VNG0058H	VNG0058H	[+]	1261.184	0.189	VNG0067H	VNG0067H	[+]	836.777	0.0858
VNG0063G	galE2	UDP-glucose 4-epimerase	1232.797	0.0855	VNG0073C	VNG0073C	[+]	824.847	0.177
VNG0065G	gmd	GDP-D-mannose dehydratase	1261.184	0.16	VNG0075H	VNG0075H	[+]	1800	0.117
VNG0090G	moeA1	Molybdenum cofactor biosynthesis protein	756.213	0.152	VNG0077H	VNG0077H	[+]	1745.69	0.184
VNG0094C	VNG0094C	[+]	1044.687	0.108	VNG0095G	gapB	Glyceraldehyde 3-phosphate dehydrogenase	1800	0.193
VNG0096C	VNG0096C	[+]	1389.13	0.1	VNG0097G	hsp2	Putative heat shock protein	1557.692	0.0313
VNG0098G	rimK	Ribosomal protein S6 modification protein	1322.069	0.0458	VNG0133G	rpa	Replication A related protein	1646.341	0.0609
VNG0116H	VNG0116H	[+]	745.914	0.13	VNG0134G	hpyA	Archaeal histone A1	1800	0.0483
VNG0121H	VNG0121H	[+]	1261.184	0.135	VNG0142C	VNG0142C	putative transcription regulator (MarR family)	1800	0.0437
VNG0127C	VNG0127C	[+]	1354.77	0.0463	VNG0186G	pepB1	Aminopeptidase homolog	1800	0.168
VNG0134G	hpyA	Archaeal histone A1	1389.13	0.112	VNG0216H	VNG0216H	[+]	801.98	0.0762
VNG0146H	VNG0146H	[+]	1232.797	0.15	VNG0219H	VNG0219H	[+]	1441.281	0.152
VNG0150H	VNG0150H	cytochrome C biogenesis protein, This protein consists of the transmembrane (i.e. non-catalytic) region of Cytochrome C biogenesis proteins also known as disulphide interchange proteins.	1322.069	0.0717	VNG0228G	guaAa	GMP synthase subunit A	1800	0.172
VNG0154G	merA	putative dihydrolipoamide Dehydrogenase	1261.184	0.19	VNG0251C	VNG0251C	[+]	1646.341	0.137
VNG0156C	VNG0156C	putative transcription regulator	1006.299	0.186	VNG0261H	VNG0261H	[+]	1646.341	0.198
VNG0157G	oxlT	Oxalate/formate antiporter	1322.069	0.154	VNG0274C	VNG0274C	[+]	1406.25	0.131
VNG0168H	VNG0168H	[+]	1232.797	0.199	VNG0284C	VNG0284C	putative Fe-S cluster-containing oxidoreductase	791.016	0.106
VNG0178H	VNG0178H	[+]	1261.184	0.0301	VNG0326G	metS	Methionine-tRNA synthetase	836.777	0.182
VNG0179C	VNG0179C	RIO1 family eukaryotic protein kinase	756.213	0.153	VNG0354C	VNG0354C	putative metal-binding membrane protease	849.057	0.107
VNG0183G	xthA	Endonuclease IV	726.136	0.132	VNG0373H	VNG0373H	[+]	1800	0.102
VNG0192G	ftsZ2	Cell division protein ftsZ homolog	1130.973	0.199	VNG0374G	nusG	Transcription termination-antitermination factor	1646.341	0.0396
VNG0213H	VNG0213H	transposase	1463.359	0.147	VNG0375G	secE	Preprotein translocase secE subunit (Protein transport protein SEC61 gamma subunit homolog)	1557.692	0.0418
VNG0222C	VNG0222C	[+]	1389.13	0.11	VNG0376G	ftsZ1	Cell division protein ftsZ	750	0.18
VNG0227H	VNG0227H	[+]	1108.092	0.0731	VNG0390G	graD1	Glucose-1-phosphate thymidylyltransferase	1694.561	0.0469
VNG0231C	VNG0231C	[+]	1261.184	0.116	VNG0401G	epf2	mRNA 3'-end processing factor homolog	1646.341	0.0874
VNG0237H	rpc10	DNA-directed RNA polymerase, 7 kDa subunit	1232.797	0.191	VNG0410G	rfbU2	LPS biosynthesis	1600.791	0.127
VNG0243C	VNG0243Cm	[+]	745.914	0.124	VNG0420H	VNG0420H	[+]	1800	0.143
VNG0249G	fbr	Copper binding proteins/plastocyanin/azurin	1389.13	0.0677	VNG0438G	ferA3	Ferredoxin	1800	0.0122
VNG0252C	VNG0252C	[+]	1065	0.143	VNG0468C	VNG0468C	putative flavoprotein	1646.341	0.087
VNG0283C	VNG0283C	molecular chaperone (small heat shock protein, Hsp20)	1179.692	0.154	VNG0469H	VNG0469H	[+]	824.847	0.179
VNG0286C	VNG0286C	probable transposase	619.386	0.164	VNG0487H	VNG0487H	[+]	1745.69	0.166
VNG0294G	pnm	N-methyltransferase homolog	1463.359	0.162	VNG0502G	aspB1	Aspartate aminotransferase	849.057	0.0854
VNG0309C	VNG0309C	2-amino-3,7-dideoxy-D-threo-hept-6-ulosonate synthase	568	0.119	VNG0509H	VNG0509H	[+]	1694.561	0.00908
VNG0319H	VNG0319H	[+]	877.346	0.147	VNG0530G	troR	Iron-dependent repressor	1694.561	0.199
VNG0320H	VNG0320H	ArsR family transcription reglator	1044.687	0.198	VNG0540G	imp	Immunogenic protein	1800	0.105
VNG0326G	metS	Methionine-tRNA synthetase	766.8	0.181	VNG0543H	VNG0543H	[+]	801.98	0.101
VNG0327G	gadD	Tyrosine decarboxylase	756.213	0.0891	VNG0548C	VNG0548C	Nucleolar RNA-binding protein	1600.791	0.142
VNG0329G	caaX	Zinc metalloproteinase homolog	756.213	0.188	VNG0549G	eif2a	Probable translation initiation factor 2 alpha subunit (eIF-2-alpha)	931.034	0.195
VNG0361C	VNG0361C	[+]	599.062	0.198	VNG0550G	rps27e	30S ribosomal protein S27e	1646.341	0.078
VNG0402H	VNG0402H	[+]	745.914	0.098	VNG0572G	dfp	FMN-binding pantothenate metabolism flavoprotein	1800	0.0335
VNG0421C	VNG0421C	[+]	891.628	0.142	VNG0578H	VNG0578H	[+]	1109.589	0.108
VNG0431G	apa	Diadenosine tetraphosphate pyrophosphohydrolase	745.914	0.198	VNG0582C			1646.341	0.164
VNG0451G	phoU	Transcriptional regulator	824.516	0.0668	VNG0586C	VNG0586C	PetE plastocyanin.	1646.341	0.0508
VNG0474G	porA	pyruvate ferredoxin oxidoreductase, subunit alpha	766.8	0.162	VNG0599C	VNG0599C	[+]	1177.326	0.0946
VNG0487H	VNG0487H	[+]	1179.692	0.198	VNG0600C	VNG0600C	[+]	1745.69	0.0582
VNG0488H	VNG0488H	[+]	766.8	0.105	VNG0610G	hhoA	4-hydroxybenzoate octaprenyltransferase	1109.589	0.125
VNG0498C	VNG0498C	[+]	756.213	0.0464	VNG0632G	purK	Phosphoribosylaminoimidazole carboxylase ATP binding subunit	1310.68	0.0704

Experiment A					Experiment B				
ORF	Gene	Function	Period	p-value	ORF	Gene	Function	Period	p-value
VNG0499G	cna	putative nucleotide methyltransferase	745.914	0.124	VNG0633G	purE	Phosphoribosylaminoimidazole carboxylase catalytic subunit	1557.692	0.117
VNG0503C	VNG0503C	putative methyltransferase.	1232.797	0.111	VNG0637G	ndhG5	NADH dehydrogenase/oxidoreductase	1600.791	0.15
VNG0507C	VNG0507C	[+]	1232.797	0.194	VNG0659H	VNG0659H	[+]	1646.341	0.178
VNG0527C	VNG0527C	[+]	891.628	0.0694	VNG0664G	birL	Biotin acetyl-CoA carboxylase ligase	813.253	0.19
VNG0537C	VNG0537C	[+]	777.688	0.124	VNG0667G	trp4	ABC transporter, ATP-binding protein homolog	1694.561	0.089
VNG0542C	VNG0542C	putative oxidoreductase	1322.069	0.172	VNG0678G	acaB1	3-ketoacyl-CoA thiolase	1745.69	0.0848
VNG0549G	eif2a	Probable translation initiation factor 2 alpha subunit (eIF-2-alpha)	1232.797	0.183	VNG0688H	VNG0688H	[+]	780.347	0.116
VNG0551G	rpl44e	50S ribosomal protein L44E	1261.184	0.137	VNG0734G	tfbB	Transcription initiation factor IIB 2 (TFIIB 2)	1600.791	0.0676
VNG0560C	VNG0560C	[+]	556.459	0.0742	VNG0750C	VNG0750C	putative GAF domain-containing protein (PF1590)	1478.102	0.178
VNG0566C	VNG0566C	[+]	1261.184	0.0805	VNG0751C	VNG0751C	putative transcription regulator (PadR family)	1557.692	0.0341
VNG0574C	VNG0574C	[+]	1261.184	0.112	VNG0758C	VNG0758C	[+]	1516.854	0.0472
VNG0583G	cyb	Cytochrome b6	1154.819	0.106	VNG0771G	aldY2	Aldehyde dehydrogenase (Retinol)	813.253	0.12
VNG0587H	VNG0587H	[+]	556.459	0.0868	VNG0782H	VNG0782H	HEAT repeat-containing protein. Related to phycobilisome proteins of cyanobacteria	1600.791	0.0567
VNG0609C	VNG0609C	[+]	756.213	0.119	VNG0811H	VNG0811H	[+]	801.98	0.161
VNG0628G	gdhA1	Glutamate dehydrogenase	766.8	0.0765	VNG0816G	chi	Chitinase	836.777	0.195
VNG0629G	aspB2	Aspartate aminotransferase	1154.819	0.136	VNG0834C	VNG0834C	[+]	916.29	0.189
VNG0639G	ndhG4	NADH dehydrogenase/oxidoreductase	1130.973	0.173	VNG0863H	VNG0863H	[+]	1800	0.0684
VNG0641C	VNG0641C	NADH-ubiquinone/plastoquinone oxidoreductase chain 6	1154.819	0.0943	VNG0869G	tfbD	Transcription initiation factor IIB 4 (TFIIB 4)	861.702	0.158
VNG0648G	ndhG3	NADH dehydrogenase/oxidoreductase	777.688	0.124	VNG0892H	VNG0892H	[+]	931.034	0.101
VNG0653G	mcmA1_2	Methylmalonyl-CoA mutase, subunit alpha	1232.797	0.124	VNG0893G	udp2	Uridine phosphorylase	1600.791	0.148
VNG0659H	VNG0659H	[+]	766.8	0.179	VNG0905G	pmu2	Phosphomannomutase	1800	0.0822
VNG0660H	VNG0660H	[+]	540	0.149	VNG0926H	VNG0926H	[+]	916.29	0.12
VNG0665G	coxB1	Cytochrome c oxidase subunit II	800.418	0.0969	VNG0940G	acs3	Acetyl-CoA synthetase (ADP forming)	836.777	0.0635
VNG0667G	trp4	ABC transporter, ATP-binding protein homolog	800.418	0.0565	VNG0987H	VNG0987H	[+]	1646.341	0.188
VNG0673G	mcmA2	Methylmalonyl-CoA mutase	906.383	0.167	VNG1041H	VNG1041H	[+]	824.847	0.0781
VNG0678G	acaB1	3-ketoacyl-CoA thiolase	1205.66	0.0955	VNG1060H	VNG1060H	[+]	888.158	0.0971
VNG0694G	nthB	Endonuclease III	1290.909	0.102	VNG1065C	VNG1065C	[+]	780.347	0.172
VNG0708H	VNG0708H	[+]	1130.973	0.198	VNG1120H	VNG1120H	[+]	740.402	0.095
VNG0711C	VNG0711C	[+]	1108.092	0.106	VNG1164C	VNG1164C	Mechanosensitive ion channel (small conductance), Mechanosensitive channels provide protection against hypo-osmotic shock, responding both to stretching of the cell membrane and to membrane depolarisation.The pressure threshold for MscS opening is 50% that of MscL (large conductance).	1646.341	0.117
VNG0713C	VNG0713C	[+]	1232.797	0.144	VNG1173G	eef1b	Elongation factor 1-beta (EF-1-beta) (aEF-1beta)	1557.692	0.156
VNG0726C	VNG0726C	putative transcription regulator (TetR family)	1290.909	0.174	VNG1190G	sod1	Superoxide dismutase [Mn] 1	1800	0.0523
VNG0727C	VNG0727C	Multi Antimicrobial Extrusion drug/sodium antiporter, These proteins mediate resistance to a wide range of cationic dyes, fluroquinolones, aminoglycosides and other structurally diverse antibodies and drugs. MATE proteins are found in bacteria, archaea and eukaryotes.	1354.77	0.138	VNG1194H	VNG1194H	[+]	1646.341	0.0562
VNG0738H	VNG0738H	[+]	1006.299	0.141	VNG1226H	VNG1226H	[+]	1694.561	0.147
VNG0750C	VNG0750C	putative GAF domain-containing protein (PF1590)	1463.359	0.0979	VNG1256G	ribG	Riboflavin-specific deaminase	824.847	0.152
VNG0757G	tfeA	Transcription initiation factor IIE alpha subunit	1290.909	0.162	VNG1263C	VNG1263C	Phosphotransferase system IIC components, glucose/maltose/N-acetylglucosamine-specific	1745.69	0.177
VNG0784G	pssA	CDP-diacylglycerol-serine O-phosphatidyltransferase	745.914	0.0585	VNG1289H	VNG1289H	[+]	1516.854	0.195
VNG0810H	VNG0810H	[+]	1108.092	0.106	VNG1291H	VNG1291H	[+]	1516.854	0.0441
VNG0835G	idr2	Iron-dependent repressor homolog	766.8	0.0559	VNG1300H	VNG1300H	[+]	780.347	0.171
VNG0864G	purL	Phosphoribosylformylglycinamidine synthase II	1179.692	0.15	VNG1370G	hemU	Iron (III) ABC transporter permease	1153.846	0.177
VNG0875C	VNG0875Cm	M50 family peptidase (metalloprotease)	756.213	0.0904	VNG1384H	VNG1384H	[+]	824.847	0.181
VNG0882G	pho2	putative phosphatase (COG match). OR putative haloacid dehalogenase-type hydrolase (PFAM and PDB matches).	877.346	0.196	VNG1410H	VNG1410H	[+]	1800	0.116
VNG0938G	gufA	GufA protein, putative divalent cation transporter (PFAM, COG matches).	745.914	0.0922	VNG1438H	VNG1438H	[+]	1800	0.174
VNG0943C	VNG0943C	[+]	1425.279	0.169	VNG1451C	VNG1451C	putative sugar-specific transcription regulator	1694.561	0.168
VNG0949G	gspE3	Type II secretion system protein	777.688	0.157	VNG1470G	pri	DNA primase , small subunit	1800	0.199
VNG0978H	VNG0978H	[+]	1086.119	0.179	VNG1482G	acd5	Acyl-CoA dehydrogenase	1745.69	0.171

Experiment A					Experiment B				
ORF	Gene	Function	Period	p-value	ORF	Gene	Function	Period	p-value
VNG0996G	boa4	Bacterio-opsin activator-like protein	1322.069	0.149	VNG1483C	VNG1483C	putative transcription regulator, Function assigned on the basis of match to COG1813: Predicted transcription factor, homolog of eukaryotic MBF1	1745.69	0.0783
VNG1005H	VNG1005H	[+]	1232.797	0.177	VNG1488G	boa2	Bacterio-opsin activator-like protein	1800	0.185
VNG1007H	VNG1007H	[+]	1261.184	0.0892	VNG1494G	rpl37e	50S ribosomal protein L37e	1557.692	0.0968
VNG1014G	polIV	DNA polymerase IV; may be involved in DNA repair	1232.797	0.0979	VNG1496G	snp	snRNP homolog	1516.854	0.168
VNG1026H	VNG1026H	[+]	580.03	0.18	VNG1497C	VNG1497C	[+]	1800	0.139
VNG1035C	VNG1035C	putative choline dehydrogenase flavoprotein/oxidoreductase	1290.909	0.103	VNG1524C	VNG1524C	[+]	1557.692	0.159
VNG1042H	VNG1042H	[+]	1322.069	0.176	VNG1525C	VNG1525C	putative accessory protein for magnesium influx, Unlike in most organisms this protein in Halobacterium NRC-1 is not in an operon with Mg transporter protein MgtB. Other proteins in this family are SapB (B. subtilis)and several hypothetical proteins.	813.253	0.143
VNG1048G	udg1	UDP-glucose dehydrogenase	1154.819	0.0823	VNG1533H	VNG1533H	[+]	1646.341	0.173
VNG1058H	VNG1058H	[+]	550.862	0.0616	VNG1574G	cobA	Cobalamin adenosyltransferase	1800	0.118
VNG1066C	VNG1066C	putative glycosyltransferase involved in polysaccharide/cell wall biosynthesis.	1261.184	0.121	VNG1580H	cobS	Cobalamin(Cbl)-5-phosphate synthase	780.347	0.198
VNG1070G	gpdA1	FAD-dependent oxidoreductase	1290.909	0.0729	VNG1581C	cobY	GTP:adenosylcobinamide (AdoCbi)-phosphate nucleotidyltransferase.	769.962	0.168
VNG1074G	ykfB2	Chloromuconate cycloisomerase	689.568	0.07	VNG1585C	VNG1585Cm	Uncharacterized conserved protein	1646.341	0.1
VNG1086C	VNG1086C	[+]	1154.819	0.158	VNG1591H	VNG1591H	[+]	1694.561	0.121
VNG1094H	VNG1094H	[+]	1205.66	0.157	VNG1595C	modA	putative molybdate transport protein, permease component of ABC transporter	1516.854	0.115
VNG1099C	VNG1099C	[+]	1322.069	0.0989	VNG1607G	cheC2	Chemotaxis protein	1557.692	0.176
VNG1101C	VNG1101C	predicted membrane protein.	1322.069	0.14	VNG1618H	VNG1618H	NADPH-dependent FMN reductase , NADPH-dependent FMN reductase (EC:1.5.1.29) reduces FMN and also reduces riboflavin and FAD, although more slowly. Members of this family catalyse the reaction: NAD(P)H + FMN = NAD(P)(+) + FMNH(2)	1310.68	0.192
VNG1105G	rpl1p	50S ribosomal protein L1P (HL8)	1290.909	0.175	VNG1619H	VNG1619H	[+]	861.702	0.0966
VNG1111G	drg	Hypothetical protein Vng1111g	777.688	0.175	VNG1637G	hcpA	Halocyanin precursor-like	555.556	0.122
VNG1130H	VNG1130H	[+]	1232.797	0.115	VNG1644G	nrdB2	Ribonucleoside reductase large chain	1800	0.177
VNG1133G	rps4p	30S ribosomal protein S4P	1354.77	0.174	VNG1721G	trkH2	TRK potassium uptake system protein	1800	0.188
VNG1150G	idsA	Geranylgeranyl diphosphate synthase	1154.819	0.153	VNG1733G	htr17	Htr17 transducer	824.847	0.119
VNG1164C	VNG1164C	Mechanosensitive ion channel (small conductance), Mechanosensitive channels provide protection against hypo-osmotic shock, responding both to stretching of the cell membrane and to membrane depolarisation.The pressure threshold for MscS opening is 50% that of MscL (large conductance).	1232.797	0.178	VNG1768G	eif5a	Translation initiation factor 5A (eIF-5A) (Hypusine-containing protein)	946.262	0.135
VNG1168C	VNG1168C	[+]	1232.797	0.138	VNG1776G	nirH	putative transcription regulator, structural match to lrp-like transcriptional regulator (e = 1x10E-28), COG1552.	1406.25	0.119
VNG1180G	msrA	Peptide methionine sulfoxide reductase msrA	1130.973	0.155	VNG1814G	carB	Carbamoyl-phosphate synthase large chain	1800	0.0981
VNG1181G	flaA1b	Flagellin A1 precursor	756.213	0.104	VNG1848H	VNG1848H	thiaminS, ThiS (thiaminS) is a 66 aa protein involved in sulphur transfer. ThiS is coded in the thiCEFSGH operon in E. coli. This family of proteins have two conserved Glycines at the C terminus. Thiocarboxylate is formed at the last G in the activation process.	961.995	0.104
VNG1185G	pqqE	Coenzyme PQQ synthesis protein	605.687	0.194	VNG1856G	htr3	Htr3 transducer	1516.854	0.131
VNG1209G	hutG	Probable formimidoylglutamase/arginase family protein	970.633	0.191	VNG1857C	VNG1857C	putative leucine binding protein, This family includes extracellular ligand binding domains of a wide range of receptors. This family also includes the bacterial amino acid binding proteins of known structure.	1478.102	0.141
VNG1220H	VNG1220H	[+]	756.213	0.0683	VNG1938C	VNG1938C	[+]	824.847	0.114
VNG1226H	VNG1226H	[+]	745.914	0.178	VNG1940H	VNG1940H	[+]	1800	0.0286
VNG1228C	VNG1228C	[+]	1354.77	0.191	VNG1944C	VNG1944C	Phosphoribosylformylglycinamidine (FGAM) synthase	1800	0.0685
VNG1252G	yhcR	Phosphoesterase	1503.529	0.0527	VNG1948H	VNG1948H	[+]	1800	0.134
VNG1283H	VNG1283H	[+]	891.628	0.199	VNG1960H	VNG1960H	[+]	679.53	0.152

Experiment A					Experiment B				
ORF	Gene	Function	Period	p-value	ORF	Gene	Function	Period	p-value
VNG1294G	slyD	FK506 binding protein (Peptidyl-prolyl cis-trans isomerase), Peptidylprolyl isomerases accelerate protein folding by catalyzing the cis-trans isomerization of proline imidic peptide bonds in oligopeptides. These proteins are found in a variety of organisms.	1354.77	0.125	VNG1963H	VNG1963H	[+]	836.777	0.186
VNG1308G	sdhB	Succinate dehydrogenase subunit B	1322.069	0.176	VNG2024H	VNG2024H	[+]	824.847	0.162
VNG1310G	sdhC	Succinate dehydrogenase hydrophobic membrane anchor protein	1179.692	0.193	VNG2032G	fad1	Enoyl-CoA hydratase	769.962	0.188
VNG1320G	cbp	Calcium-binding protein homology	1389.13	0.11	VNG2037C	VNG2037C	putative sensory histidine kinase, contains strong COG and PFAM hits to N-Term PAS/PAC and c-term sensory histidine kinase. Strong structure hit (2X10E-109) to FixL of R. meliloti	1600.791	0.0689
VNG1336C	VNG1336C	[+]	766.8	0.0852	VNG2053G	rpoE'	DNA-directed RNA polymerase subunit E'	1646.341	0.133
VNG1375C	VNG1375C	[+]	921.635	0.118	VNG2056G	eif2g	Probable translation initiation factor 2 gamma subunit (eIF-2-gamma)	1800	0.19
VNG1381H	VNG1381H	[+]	745.914	0.0233	VNG2078G	hat2	Probable acetyltransferase	1153.846	0.186
VNG1412H	VNG1412H	[+]	1154.819	0.0945	VNG2093G	glnA	Glutamine synthetase	1800	0.0224
VNG1429C	VNG1429C	[+]	756.213	0.184	VNG2094G	trh4	Transcription regulator, PFAM1037:AsnC/Lrp family of transcription regulators\nCOG1522:Transcription regulators (PET = 72)	1646.341	0.0679
VNG1470G	pri	DNA primase , small subunit	756.213	0.101	VNG2122G	ilvE2	Branched-chain amino acid aminotransferase	1800	0.174
VNG1494G	rpl37e	50S ribosomal protein L37e	1290.909	0.153	VNG2147G	hmp	Membrane protein	1441.281	0.071
VNG1529G	mmdA	Methylmalonyl-CoA decarboxylase, subunit alpha	745.914	0.168	VNG2157C	VNG2157C	[+]	1600.791	0.0237
VNG1538H	VNG1538H	[+]	1179.692	0.173	VNG2162C	VNG2162C	[+]	1600.791	0.14
VNG1540G	ywfD	Glucose 1-dehydrogenase	1261.184	0.142	VNG2165H	VNG2165H	[+]	1800	0.195
VNG1544G	clc	Chloride channel	1290.909	0.156	VNG2177H	VNG2177H	[+]	1800	0.134
VNG1547C	VNG1547C	[+]	1290.909	0.136	VNG2183H	VNG2183H	[+]	836.777	0.16
VNG1557G	cbiH2	precorrin-3 C-17 methyltransferase Cobalamin biosynthesis	1179.692	0.19	VNG2190G	ileS	Isoleucyl-tRNA synthetase	1646.341	0.102
VNG1559H	VNG1559H	[+]	1130.973	0.142	VNG2197H	VNG2197H	[+]	1310.68	0.0727
VNG1574G	cobA	Cobalamin adenosyltransferase	745.914	0.0733	VNG2199H	VNG2199H	[+]	1557.692	0.0181
VNG1577C	VNG1577C	[+]	1232.797	0.156	VNG2214G	[+]	multidrug efflux pump, Strong hits: PF1544 MatE domain containing protein. Also strong hits to COG0534 (Multidrug efflux pump) MatE (Multiple Antimicrobial Exclusion) domains mediate resistance to many antimicrobial agents and typically function as drug/sodium antiporters.	961.995	0.0745
VNG1585C	VNG1585Cm	Uncharacterized conserved protein	1590.871	0.177	VNG2226G	cctA	Thermosome alpha subunit (Thermosome subunit 1) (Chaperonin alpha subunit)	1800	0.0505
VNG1608C	VNG1608C	[+]	745.914	0.12	VNG2236H	VNG2236H	[+]	849.057	0.0506
VNG1609C	VNG1609C	[+]	540	0.199	VNG2246H	VNG2246H	[+]	849.057	0.156
VNG1613H	VNG1613H	[+]	745.914	0.0395	VNG2281C	VNG2281C	[+]	1694.561	0.196
VNG1621H	VNG1621H	[+]	1290.909	0.0349	VNG2317G	cbiO1	Cobalt transport ATP-binding protein	801.98	0.098
VNG1631G	cbiO2	Cobalt transport ATP-binding protein	1232.797	0.109	VNG2321G	ydaF	Putative acetyltransferase	1800	0.173
VNG1632G	cbiQ	Cobalt transport protein	1108.092	0.121	VNG2343G	ykfD	Oligopeptide ABC transporter ATP-binding	1800	0.15
VNG1640H	VNG1640H	ATPase subunit of protease ClpXt / PDB 1DPU C-terminal domain of replication protein A COG1219.	1261.184	0.156	VNG2366C	VNG2366C	[+]	1646.341	0.0951
VNG1664H	VNG1664H	[+]	1154.819	0.14	VNG2383G	nrdA	Ribonucleoside reductase small chain	1800	0.167
VNG1667G	cdc48c	CdcH protein	788.889	0.196	VNG2387H	VNG2387H	[+]	801.98	0.0595
VNG1678H	VNG1678H	[+]	1290.909	0.108	VNG2394G	tssB	Thiosulfate sulfurtransferase	1745.69	0.023
VNG1740C	VNG1740C	[+]	1322.069	0.18	VNG2411G	orc7	Orc / cell division control protein 6	1516.854	0.169
VNG1754G	phr1	Photolyase/cryptochrome	1290.909	0.119	VNG2420G	metA	Probable homoserine O-acetyltransferase	801.98	0.115
VNG1758H	VNG1758H	[+]	545.377	0.121	VNG2423G	serB	Phosphoserine phosphatase	1646.341	0.013
VNG1775C	VNG1775C	Siroheme Biosynthesis Protein, A Bifunctional Nad-Dependent Dehydrogenase and Ferrochelatase Involved In Siroheme Synthesis.	1389.13	0.0992	VNG2431C	VNG2431C	[+]	1745.69	0.135
VNG1785G	panF	Pantothenate permease	735.893	0.093	VNG2446H	VNG2446H	[+]	931.034	0.0732
VNG1788C	VNG1788C	[+]	756.213	0.106	VNG2482G	pstB1	Phosphate ABC transporter ATP-binding	861.702	0.161
VNG1816G	trh3	Transcription regulator	756.213	0.0889	VNG2488C	VNG2488C	[+]	836.777	0.179
VNG1851G	suk	Sugar kinase	756.213	0.101	VNG2490H	VNG2490H	[+]	813.253	0.16
VNG1903C	VNG1903Cm	predicted transcriptional regulator, exact copy of VNG1886C	788.889	0.119	VNG2526G	dppF	Dipeptide ABC transporter ATP-binding	1646.341	0.0947
VNG1917H	VNG1917H	[+]	756.213	0.135	VNG2529G	dppB2	Dipeptide ABC transporter permease	1478.102	0.136

Experiment A

ORF	Gene	Function	Period	p-value
VNG1940H	VNG1940H	[+]	1290.909	0.18
VNG1943H	VNG1943H	[+]	1086.119	0.152
VNG1997G	infB	Probable translation initiation factor IF-2	906.383	0.189
VNG2008H	VNG2008H	[+]	1108.092	0.112
VNG2014H	VNG2014H	[+]	1389.13	0.14
VNG2028H	VNG2028H	[+]	1261.184	0.0888
VNG2036G	hlx1	putative response regulator, COG0784:cheY-like receiver domain\nPFAM00072:response regulator receiver domain	1261.184	0.171
VNG2043G	ham1	HAM1 protein homolog	1086.119	0.11
VNG2047G	rps27ae	30S ribosomal protein S27E	1590.871	0.167
VNG2048G	rps24e	30S ribosomal protein S24e	1205.66	0.145
VNG2059H	VNG2059H	[+]	756.213	0.073
VNG2065G	dgs	Dolichol-P-glucose transferase	1354.77	0.0616
VNG2067H	VNG2067H	[+]	735.893	0.191
VNG2068C	VNG2068C	[+]	1261.184	0.119
VNG2084G	phnE	Transport protein	1205.66	0.0736
VNG2091H	VNG2091H	putative phosphatase	745.914	0.0588
VNG2097C	VNG2097C	[+]	1290.909	0.171
VNG2101H	VNG2101H	[+]	1425.279	0.196
VNG2121C	VNG2121C	[+]	1205.66	0.162
VNG2128C	VNG2128C	[+]	1290.909	0.0556
VNG2130G	minD2	Cell division inhibitor	1354.77	0.0798
VNG2133H	VNG2133H	[+]	1154.819	0.156
VNG2154C	VNG2154C	[+]	1261.184	0.184
VNG2156C	VNG2156C	[+]	735.893	0.189
VNG2157C	VNG2157C	[+]	1086.119	0.175
VNG2162C	VNG2162C	[+]	1086.119	0.13
VNG2191H	VNG2191H	[+]	599.062	0.149
VNG2208G	trpS1	Tryptophanyl-tRNA synthetase	1205.66	0.187
VNG2213G	brr2	Pre-mRNA splicing helicase	745.914	0.0828
VNG2216G	lip	Probable lipoic acid synthetase (Lip-syn) (Lipoate synthase)	1108.092	0.167
VNG2220G	lpdA	Dihydrolipoamide dehydrogenase	1154.819	0.181
VNG2226G	cctA	Thermosome alpha subunit (Thermosome subunit 1) (Chaperonin alpha subunit)	1108.092	0.18
VNG2237G	tyrS	Tyrosyl-tRNA synthetase	1086.119	0.151
VNG2247G	hisG	ATP phosphoribosyltransferase	1290.909	0.0432
VNG2249G	trzA	N-ethylammeline chlorohydrolase	1389.13	0.179
VNG2251G	achY	Adenosylhomocysteinase	1108.092	0.184
VNG2270G	mutS3	Mismatch repair protein	756.213	0.0658
VNG2273H	VNG2273H	[+]	1354.77	0.049
VNG2281C	VNG2281C	[+]	1425.279	0.115
VNG2292H	VNG2292H	[+]	921.635	0.131
VNG2304H	VNG2304H	[+]	877.346	0.179
VNG2335H	VNG2335H	[+]	756.213	0.1
VNG2338G	polA2	DNA polymerase II large subunit	735.893	0.0688
VNG2353H	VNG2353H	[+]	756.213	0.136
VNG2369C	VNG2369C	[+]	921.635	0.148
VNG2378G	nosF1	Copper transport ATP-binding protein	1130.973	0.195
VNG2398G	scm	24-sterol C-methyltransferase	1025.134	0.171
VNG2411G	orc7	Orc / cell division control protein 6	756.213	0.119
VNG2415H	VNG2415H	[+]	745.914	0.119
VNG2432C	VNG2432C	[+]	1086.119	0.172
VNG2437G	argG	Argininosuccinate synthetase	1290.909	0.188
VNG2470C	VNG2470C	[+]	745.914	0.168
VNG2477H	VNG2477H	[+]	756.213	0.0577
VNG2484G	pstC1	Phosphate transporter permease	812.288	0.0679
VNG2486G	yqgG	Phosphate ABC transporter binding	800.418	0.159

Experiment B

ORF	Gene	Function	Period	p-value
VNG2531G	dppC1	Dipeptide ABC transporter permease	1406.25	0.191
VNG2537G	entB	Isochorismatase	695.876	0.192
VNG2543C	VNG2543C	[+]	1600.791	0.0228
VNG2544H	VNG2544H	[+]	836.777	0.109
VNG2580C	VNG2580C	[+]	1478.102	0.198
VNG2585H	VNG2585H	[+]	1557.692	0.0876
VNG2599H	VNG2599H	Sec61beta subunit, This a component of the Sec61/SecYEG protein secretory system found in eukaryotes and archaea and is possibly homologous to the bacterial SecG.	1694.561	0.0161
VNG2602G	arsB	Arsenite transport protein	813.253	0.0868
VNG2612G	rli	RNase L inhibitor homolog	1557.692	0.127
VNG2617G	adh2	Alcohol dehydrogenase	1694.561	0.199
VNG2626H	VNG2626H	[+]	1646.341	0.181
VNG2633H	VNG2633H	[+]	836.777	0.13
VNG2640G	gcvT2	Aminomethyltransferase	1600.791	0.0351
VNG2646C	VNG2646C	[+]	1646.341	0.137
VNG2653C	VNG2653C	putative transposase	836.777	0.149
VNG2665G	rpoB'	DNA-directed RNA polymerase subunit B'	888.158	0.106
VNG5009H	VNG5009H	transcription regulator, This family of DNA binding helix-turn helix proteins includes a bacterial plasmid copy control protein, bacterial methylases, various bacteriophage transcription control proteins.\n\n	1557.692	0.0567
VNG5061C	VNG5061C	[+]	759.85	0.11
VNG5073H	VNG5073H	[+]	801.98	0.17
VNG5091C	VNG5091C	[+]	769.962	0.143
VNG5100C	VNG5100C	[+]	836.777	0.0696
VNG5106H	VNG5106H	[+]	849.057	0.154
VNG5108H	VNG5108H	[+]	824.847	0.141
VNG5116H	VNG5116H	[+]	1800	0.163
VNG5131H	VNG5131H	[+]	916.29	0.188
VNG5142G	tbpC	transcription initiation factor IID	801.98	0.118
VNG5144H	VNG5144H	Transcriptional regulator PadR-like family	1516.854	0.0804
VNG5149H	VNG5149H	Putative ISH4 transposase (VNG0918H)	824.847	0.0701
VNG5160H	VNG5160H	[+]	813.253	0.171
VNG5163G	tbpD	TATA-box binding protein D	801.98	0.162
VNG5168H	VNG5168H	[+]	801.98	0.0402
VNG6135C	VNG6135C	[+]	1646.341	0.0462
VNG6148H	VNG6148H	predicted transposase	813.253	0.175
VNG6150G	orc1	Orc / cell division control protein 6	824.847	0.102
VNG6155H	VNG6155H	[+]	1341.06	0.156
VNG6157H	VNG6157H	[+]	836.777	0.168
VNG6166H	VNG6166H	[+]	836.777	0.0466
VNG6168H	VNG6168H	[+]	849.057	0.136
VNG6177G	kdpB	Potassium-transporting ATPase B chain	824.847	0.107
VNG6183C	VNG6183C	[+]	836.777	0.166
VNG6189H	VNG6189H	[+]	801.98	0.154
VNG6196G	phoT2	Sodium-dependent phosphate transporter	791.016	0.0815
VNG6197H	VNG6197H	[+]	824.847	0.103
VNG6203H	VNG6203H	[+]	836.777	0.052
VNG6230G	gvpK2	GvpK protein 2	813.253	0.0924
VNG6270G	gldA	Sn-glycerol-1-phosphate dehydrogenase	1745.69	0.0455
VNG6293C	VNG6293C	[+]	824.847	0.12
VNG6301G	aph	Alkaline phosphatase	961.995	0.127
VNG6306C	VNG6306C	[+]	824.847	0.0399
VNG6308G	gltP	Proton/sodium-glutamate symport protein	1800	0.175
VNG6309G	pyrB	Aspartate carbamoyltransferase	1646.341	0.15
VNG6332H	VNG6332H	[+]	1088.71	0.154
VNG6334H	VNG6334H	[+]	849.057	0.109
VNG6339H	VNG6339H	[+]	813.253	0.174
VNG6340H	VNG6340H	[+]	824.847	0.156

Experiment A

ORF	Gene	Function	Period	p-value
VNG2510H	VNG2510H	[+]	1354.77	0.173
VNG2516C	VNG2516C	putative sugar kinase	1354.77	0.155
VNG2519H	VNG2519H	[+]	1261.184	0.0568
VNG2551G	fhuG	Ferrichrome ABC transporter permease	1322.069	0.115
VNG2553G	yqeC	6-phosphogluconate dehydrogenase	1290.909	0.188
VNG2589C	VNG2589C	[+]	1290.909	0.168
VNG2619H	VNG2619H	[+]	777.688	0.0703
VNG2638G	bchP	putative flavoprotein	745.914	0.175
VNG2639G	uae	UDP-N-acetylglucosamine 2-epimerase	1354.77	0.0591
VNG2642H	VNG2642H	[+]	1130.973	0.113
VNG2647G	vacB	Ribonuclease II family protein	1354.77	0.167
VNG2652H	VNG2652H	[+]	1261.184	0.0604
VNG2664G	rpoA	DNA-directed RNA polymerase subunit A	788.889	0.188
VNG2669G	cyo	Cytochrome oxidase subunit I homolog	1232.797	0.144
VNG2677H	VNG2677H	[+]	1425.279	0.094
VNG5025G	gvpH1	GvpH protein, cluster A	777.688	0.144
VNG5048H	VNG5048H	[+]	906.383	0.142
VNG5055G	cydA	cytochrome d oxidase chain I	788.889	0.101
VNG5059C	VNG5059C	[+]	800.418	0.164
VNG5064H	VNG5064H	[+]	1290.909	0.152
VNG5066G	phoT1	Inorganic phosphate transport protein	756.213	0.185
VNG5071C	VNG5071C	sugar (and other) transporter	1545.968	0.0962
VNG5078G	trh	thioredoxin reductase-like protein	1322.069	0.154
VNG5080H	VNG5080H	[+]	1290.909	0.18
VNG5093C	VNG5093C	[+]	745.914	0.0813
VNG5098C	VNG5098C	[+]	953.731	0.149
VNG5115H	VNG5115H	[+]	891.628	0.0891
VNG5124H	VNG5124H	[+]	756.213	0.137
VNG5129H	VNG5129H	[+]	906.383	0.0842
VNG5131H	VNG5131H	[+]	850.111	0.144
VNG5139C	VNG5139C	[+]	735.893	0.188
VNG5148H	VNG5148H	[+]	891.628	0.0937
VNG5156H	VNG5156H	potential transcriptional regulator (repressor), COG1552: transcriptional regulators (PET=15)\nRosetta predicts strong similarity to DtxR repressor family	906.383	0.2
VNG5160H	VNG5160H	[+]	906.383	0.0803
VNG5166H	VNG5166H	[+]	756.213	0.167
VNG5185H	VNG5185H	[+]	745.914	0.114
VNG6133H	VNG6133H	[+]	745.914	0.113
VNG6145H	VNG6145H	[+]	745.914	0.127
VNG6148H	VNG6148H	predicted transposase	891.628	0.198
VNG6156H	VNG6156H	[+]	756.213	0.186
VNG6158H	VNG6158H	[+]	906.383	0.183
VNG6171H	VNG6171H	[+]	716.636	0.154
VNG6173C	VNG6173C	[+]	1503.529	0.166
VNG6178G	kdpC	Potassium-transporting ATPase C chain	1463.359	0.192
VNG6208C	VNG6208C	[+]	953.731	0.116
VNG6210G	gabT	Gamma-aminobutyrate aminotransferase	756.213	0.0614
VNG6220G	cat2	Cationic amino acid transporter	1425.279	0.147
VNG6255C	VNG6255C	[+]	1425.279	0.195
VNG6299G	hypE	Hydrogenase expression/formation protein	1261.184	0.0898
VNG6309G	pyrB	Aspartate carbamoyltransferase	1290.909	0.0911
VNG6315G	arcB	Ornithine carbamoyltransferase	1108.092	0.0872
VNG6320C	VNG6320C	[+]	1463.359	0.102
VNG6330H	VNG6330H	[+]	1354.77	0.163
VNG6334H	VNG6334H	[+]	1322.069	0.0723
VNG6335H	VNG6335H	[+]	756.213	0.0362
VNG6347H	VNG6347H	[+]	745.914	0.173
VNG6348H	VNG6348H	[+]	756.213	0.123
VNG6364H	VNG6364H	[+]	735.893	0.177
VNG6365H	VNG6365H	[+]	745.914	0.0664

Experiment B

ORF	Gene	Function	Period	p-value
VNG6362G	polB2	DNA polymerase B2	801.98	0.149
VNG6393H	VNG6393H	[+]	813.253	0.186
VNG6424H	VNG6424H	[+]	861.702	0.0866
VNG6438G	tbpF	TATA-box binding protein F (TATA-box factor F) (TATA sequence-binding protein F) (TBP F) (Box A binding protein F) (BAP F)	849.057	0.0987

Experiment A					Experiment B				
**ORF**	**Gene**	**Function**	**Period**	**p-value**	**ORF**	**Gene**	**Function**	**Period**	**p-value**
VNG6368H	VNG6368H	[+]	1290.909	0.0859					
VNG6381H	VNG6381H	[+]	756.213	0.112					
VNG6385H	VNG6385H	[+]	1261.184	0.195					
VNG6408G	phzF	Phenazine biosynthetic protein	1290.909	0.12					
VNG6412H	VNG6412H	[+]	716.636	0.189					
