# Supplementary material for: Diurnally Entrained Anticipatory Behavior in Archaea
Source: PLoS One. 2009 May 8;4(5):e5485. doi: 10.1371/journal.pone.0005485 (PMC2675056; doi:10.1371/journal.pone.0005485)
Supplement: Table S3 — The number of genes and average period in each of the clusters presented in Figure 3 of the Experiment A.main text. The number of genes correlated to high or low oxygen (taken from Schmid et al. 2007) are also given. (0.13 MB PDF) [file pone.0005485.s004.pdf]

Table S3. The number of genes and average period in each of the clusters presented in Figure 3 of the Experiment A.main text. The number of genes correlated to high or low oxygen (taken from Schmid et al. 2007) are also given.

Cluster	Color*	Avg. period	# genes	+ O_2 correlation	- O_2 correlation
1	orange	13.2	70	0	33 (47%)
2	red	21.1	65	0	17 (26%)
3	blue	18.5	45	20 (44%)	0
